# Clinical-Pathological Conference Series from the Medical University of Graz: Case No 164: A 46-year-old man with abdominal pain, dyspnea and rapidly progressing multiorgan failure

**DOI:** 10.1007/s00508-021-01841-y

**Published:** 2021-04-19

**Authors:** Elisabeth Fabian, Christoph Wenisch, Florian Eisner, Tina Muhr, Philipp K. Bauer, Kurt Prein, Urša Maierhofer, Sigurd F. Lax, Robert Krause, Gernot Zollner, Wolfgang Weihs, Guenter J. Krejs

**Affiliations:** 1 Division of Gastroenterology and Hepatology, Department of Internal Medicine III, Medical University of Vienna, Vienna, Austria; 2 4th Department of Internal Medicine with Infectious and Tropical Medicine, State Hospital Klinik Favoriten, Vienna, Austria; 3 Division of Emergency Medicine, Department of Internal Medicine, Medical University of Graz, Graz, Austria; 4 Division of Cardiology, Department of Internal Medicine, State Hospital (LKH) Graz II, Graz, Austria; 5 Division of Infectious Diseases and Tropical Medicine, Department of Internal Medicine I, Medical University of Vienna, Vienna, Austria; 6 Department of Pathology, State Hospital (LKH) Graz II, Graz, Austria; 7 Section of Infectious Diseases and Tropical Medicine, Department of Internal Medicine, Medical University of Graz, Graz, Austria; 8 Division of Gastroenterology and Hepatology, Department of Internal Medicine, Medical University of Graz, Auenbruggerplatz 15, 8036 Graz, Austria

**Keywords:** Leptospirosis, Weil’s disease, Pseudoacute abdomen, Myocarditis, Renal failure

## Presentation of case

### Dr. T. Muhr:

This is a case of a 46-year-old patient from France who was working as an IT coach and network supervisor. Nine days before his trip to Graz, Austria, he had taken a walk with his 8‑year-old son in the surroundings of Strasbourg, in the Alsace region. During this tour, he suffered a small wound (probably due to an insect bite) on his left forearm. Two days before he came to Graz, both the patient and his son had had a sore throat and fever for one day. On the day he left France, he made a ham and cheese sandwich, which he had also consumed the following day. On his 3rd day in Graz, the patient complained of abdominal discomfort and pain for which he took diclofenac. Except for this drug, he had not taken any other medication. The history of the patient was negative for allergies, cigarette smoking, alcohol and drug abuse; he did not have any pets. On the 4th day in Graz, his medical condition worsened; he suffered from increasing abdominal pain, two episodes of diarrhea (but no vomiting) and dyspnea, and collapsed in his hotel room.

The patient was admitted to the emergency room (ER) in a state of shock; his blood pressure was 95/45 mmHg, pulse rate 99 beats per minute, oxygen saturation 86% at room air, body temperature 36.8 °C. His skin was marbled, cyanotic and his back was livid; however, there was no rash or jaundice. Further clinical examination revealed tachycardia with rhythmic heart sounds and no murmurs, and vesicular breathing sounds in both lungs. The patient presented with abdominal guarding, diffuse abdominal pain most accentuated in the lower quadrants, livid limbs and blue acra. Electrocardiogram showed ST-elevation in leads V1–V3 and V6; echocardiography revealed significantly reduced left ventricular function and diffuse hypokinesia; there was no hemodynamically relevant valve abnormality. Coronary angiography was performed immediately and revealed a slow flow in all vessels, but no hemodynamically relevant stenosis. Since fulminant myocarditis and septic shock of unknown origin were suspected, blood cultures were taken three times and antibiotic treatment with meropenem was initiated. Computed tomography (CT) of the brain was unremarkable; CT of the abdomen showed fatty liver and one enlarged lymph node (1.4 × 0.9 cm) located above the celiac trunk.

Laboratory data on admission: Hemoglobin 13.2 g/dL (normal: 14.0–18.0 g/dL), leukocytes 2.45 G/L (normal: 4.0–9.0 G/L), 15% lymphocytes, 81% neutrophils, platelets 210 G/L (normal: 140–400 G/L), prothrombin time 48% (normal: 70–130%), creatine kinase (CK)-MB 28 U/L (normal: 1–24 U/L), high-sensitivity troponin I (hs-TnI) 95 ng/L (normal: <26 ng/L), creatinine 4.5 mg/dL (normal: 0.7–1.2 mg/dL), glomerular filtration rate (GFR) 14 mL/min/1,73m^2^ (normal: 80–140 mL/min/1,73m^2^), C‑reactive protein (CRP) 32 mg/dL (normal: <0.5 mg/dL), D‑dimer 4491 µg/L (normal: < 500 µg/L), myoglobin 241 mg/mL (normal: 23–70 mg/mL), total bilirubin 2.3 mg/dL (normal: 0.1–1.2 mg/dL), alanine aminotransferase (ALT) 327 U/L (normal: 10–50 U/L), lactate dehydrogenase (LDH) 433 U/L (normal: 120–240 U/L), procalcitonin 90 ng/mL (normal: <0.5 ng/mL). Blood cultures were reported as negative. Polymerase chain reaction (PCR) in blood was negative for meningococci, pneumococci, *Haemophilus influenzae, Staphylococcus aureus*, streptococci group B and *Listeria monocytogenes*. Blood smear was negative for fragmentocytes, haptoglobin was 266/dL (normal: 30–200/dL). Despite intensive care including artificial ventilation at maximum oxygenation, administration of inotropes (dobutamine), vasopressors (norepinephrine and vasopressin), hydrocortisone (because of massive hemodynamic instability despite inotropes and vasopressor treatment) and antibiotics (additional levofloxacin and ampicillin), the patient did not become hemodynamically stable (pO_2_ 59 mmHg, arterial pH 7.3, base excess –12, lactate > 10 mmol/L). Extracorporeal membrane oxygenation was not available. Less than 24 h after admission, the patient died of multiorgan failure.

An autopsy was performed and a diagnostic test result became available three days later.

## Differential diagnosis

### Dr. C. Wenisch:

This is a complex case of a patient with an interesting medical history. Indeed, some aspects remain unclear from the protocol. These include a questionable causality between the current condition and (1) the mentioned wound or insect bite on the left forearm of the patient debatably associated with the occurrence of a sore throat and fever one day later and (2) the consumption of a ham and cheese sandwich, and the subsequent development of abdominal discomfort and pain, both suggesting an infectious etiology. The patient was admitted to hospital with shock. Electrocardiogram revealed ST-elevation and echocardiography demonstrated significantly reduced left ventricular function and diffuse hypokinesia; coronary angiography showed a slow flow rate in all vessels, but no hemodynamically relevant stenosis. These alterations together with the finding of increased serum levels of hs-TnI, CK-MB and inflammation markers led to the diagnosis of myocarditis. This condition can be due to a variety of infectious and noninfectious causes reviewed elsewhere [1] and may even be fatal in the case of fulminant cardiac failure. Establishing the underlying etiologic pathogen of myocarditis is of utmost importance as it may alter disease management. Myocarditis is most commonly of viral etiology (e.g. coxsackie B virus, cytomegalovirus, parvovirus B19, Epstein-Barr virus), but might also be caused by a large group of bacterial pathogens [2].

Some bacterial infections are well-known to have the potential of causing fatal outcome (septic shock) even in previously healthy young persons. These include staphylococcal toxic shock syndrome, group A streptococcal toxic shock syndrome, group A streptococcal myocarditis, leptospirosis, gram-negative sepsis, typhoid fever, rickettsiosis, and infection with meningococci or *Streptococcus pneumoniae* [3].

In this patient, gram-negative sepsis and infection with meningococci or *Streptococcus pneumoniae* can be ruled out because of negative blood cultures and bacterial PCR tests. Since there is no history of “pea soup” liquid diarrhea, typhoid fever can also be excluded as the underlying cause of the patient’s condition.

Indeed, there are two conspicuous facts in the patient’s history: (1) A sore throat and fatal myocarditis, and (2) a homemade sandwich (with ham and cheese) and diarrhea. Given the sore throat and myocarditis, infection with *Corynebacterium diphtheriae* should be considered, even though the incidence of diphtheria in developed countries has been declining following effective immunization programs since the 1920s [4–6]. In the USA, only 55 cases of diphtheria were documented from 1980 through 2011, with only five cases being reported since 2000. Most cases occurred in nonimmunized or inadequately immunized persons; however, diphtheria continues to occur in other parts of the world. In the 1990s, a major epidemic was reported in all newly independent states of the former Soviet Union with more than 157,000 cases and more than 5000 deaths. In some of these countries, up to 80% of cases affected adults. Globally, reported cases of diphtheria have declined from 11,625 in 2000 to 4880 cases in 2011 [7]. The most common sites of diphtheria infections are the pharynx and the tonsils resulting in sore throat, anorexia and low-grade fever. Within 2–3 days, a bluish-white membrane forms and extends, which can vary in size from covering a small patch on the tonsils to covering most of the soft palate. This pseudomembrane firmly adheres to the tissue and attempts to remove it forcibly may cause bleeding. Extensive formation of pseudomembranes may result in airway obstruction. Patients with severe disease may further develop marked edema of the submandibular areas and the anterior neck along with lymphadenopathy, giving a characteristic “bull neck” appearance [7]. The most frequent complication in diphtheria and the most important predictor of mortality is myocarditis [8], which may occur early in the course of the disease or weeks later and can lead to heart failure [7].

Given the consumption of a homemade ham and cheese sandwich and subsequent diarrhea, various food-borne infections should be addressed as well. These include infection with salmonella, *Toxoplasma gondii* and *Listeria monocytogenes,* which are frequently associated with ham or cheese as a potential vector; however, taking into account that the incubation time of such diseases is longer than the onset of abdominal discomfort and diarrhea reported in this patient, and the fact that there were only two bouts of diarrhea, diarrhea in the context of sepsis is much more likely than diarrhea caused by a food-borne infection.

This leaves the following four infectious diseases in the differential diagnosis, which have the potential to cause death even in previously healthy young persons: (1) Rickettsial infection, (2) staphylococcal toxic shock syndrome, (3) group A streptococcal toxic shock syndrome and group A streptococcal myocarditis, and (4) leptospirosis.

Rickettsial organisms are a diverse group of gram-negative, obligately intracellular bacteria with variable pathogenicity. All species that are known to cause human disease depend on an arthropod vector (up to 24% of terrestrial arthropods carry rickettsial endosymbionts [9]) such as fleas, ticks, mites or lice [10]. According to serologic and genomic tests, the pathogens of human rickettsioses can be categorized as (1) spotted fever group rickettsiae (*R. rickettsii*: Rocky Mountain spotted fever*, R. montanensis, R. amblyommii, R. parkeri*), (2) translational group rickettsiae (rickettsiae belonging to the spotted fever group serologically, but genetically overlap with typhus group rickettsiae; these are rickettsiae with a much milder clinical course and no reported fatalities, such as *R. felis* and *R. akari* [11]) and (3) typhus group rickettsiae (*R. typhi*: murine typhus, also known as endemic or flea-borne typhus and *R. prowazekii*: endemic typhus, Brill-Zinsser disease) [10]. Moreover, diseases such as the Mediterranean spotted fever, and scalp eschar and neck lymphadenopathy (SENLAT), which is also known as *Dermacentor*-borne necrosis erythema lymphadenopathy (DEBONEL) or tick-borne lymphadenopathy (TIBOLA), are due to *R. conorii* [12] and *R. raoultii* [13]. Clinically significant rickettsial infections present with fever, headache and a rash with or without eschar; symptoms such as myalgia and arthralgia may occur. The clinical course of rickettsiosis is highly variable ranging from self-limiting in the case of *R. akari* [10] to myocarditis [14], fulminant organ failure and death due to infection with *R. rickettsii* [10]. The exact mechanisms by which rickettsia exerts the pathogenic effects on humans remain elusive. During infection, bacteria disseminate to endothelial cells which become dysfunctional and lose their integrity. Microvascular thrombosis and increased permeability of microvasculature with subsequent edema are common histologic findings in rickettsial infections [10]. Since the discussed patient did not present with a rash, the diagnosis of rickettsiosis is quite unlikely; however, another tick-borne disease presenting with fever (97% of cases), headache (81%), myalgia (68%), malaise (84%) as well as leukopenia and thrombopenia, but less frequently with gastrointestinal involvement (nausea, vomiting, diarrhea 25–68%) and only rarely with a rash (6%) is ehrlichiosis. Ehrlichiae are small, obligately intracellular bacteria with a gram-negative type cell wall that grow in cytoplasmic vacuoles to form clusters called morulae. Two distinct *Ehrlichia* species (*E. chaffeensis* and *E. ewingii*) and one *Anaplasma* species (*A. phagocytophilia*) can cause severe infection in humans. While infection with *E. chaffeensis *results in human monocytotropic ehrlichiosis, *E. ewingii* and *A. phagocytophilia* infect cells of the myeloid lineage, particularly neutrophils [15]. Ehrlichiosis may cause left ventricular dilatation and dysfunction [16] and in some cases may be fatal due to rapidly progressive myocarditis and multiorgan failure as reported for *E. chaffeensis* in a previously healthy adolescent [17]. Since the clinical course of ehrlichiosis is typically associated with fever, which was only reported for one day in the discussed patient, this diagnosis seems unlikely in this case.

Exclusion of rickettsial infections in this case reduces potential candidates in the differential diagnosis to staphylococcal or group A streptococcal toxic shock syndrome, group A streptococcal myocarditis and leptospirosis. Epidemiologic investigations demonstrated that 50–70% of cases of staphylococcal toxic shock syndrome are associated with menstruation and the use of highly absorbent tampons. Evidence of *Staphylococcus aureus* infection is not a prerequisite for the development of the disease. The staphylococcal toxic shock syndrome results from the elaboration of an enterotoxin or the structurally related enterotoxin-like toxic shock syndrome toxin-1 (TSST‑1), which causes more than 90% of menstrual cases. Nonmenstrual cases are predominantly caused by enterotoxins [15]. TSST‑1 is a superantigen produced by 5–25% of *Staphylococcus aureus* isolates [18] that binds primarily to the alpha-chain of the class II major histocompatibility complex exclusively through a low-affinity (or generic) binding site on the surface antigen N‑terminal domain. This stimulates human T cells that express VB 2, which results in the expansion of both CD4 and CD8 subsets of T lymphocytes releasing large amounts of interleukins 1 and 2, and tumor necrosis factor [19]. A staphylococcal toxic shock syndrome typically begins with nonspecific flu-like symptoms including fever, hypotension and erythroderma of variable intensity. Mucosal involvement such as conjunctival hyperemia is also common. The clinical course may rapidly progress to symptoms such as vomiting, diarrhea, confusion, myalgia and abdominal pain, reflecting the multisystemic nature of the disease. Desquamation of the skin can be observed one or two weeks after the onset of the disease. Since the discussed patient did not present with the described symptoms, the diagnosis of staphylococcal toxic shock syndrome seems unlikely in this case [20].

Lancefields’s group A streptococci consist of a single species, *Streptococcus pyogenes*, which is an organism associated with a variety of suppurative infections. Group A streptococci produce a large number of extracellular products such as streptolysins S and O, streptokinase, DNases, protease and pyrogenic exotoxins A, B and C, that may be important in local and systemic toxicity and in the spread of infection through tissue [15]. Pyrogenic exotoxins cause the rash of scarlet fever, and have been linked to severe invasive infections, including necrotizing fasciitis and a systemic syndrome termed the streptococcal toxic shock syndrome (mortality rate up to 60%) [21, 22]. After bacterial inoculation, which frequently occurs via a small wound, the incubation period is 1–4 days. Intake of nonsteroidal anti-inflammatory drugs (NSAIDs) is known to worsen the course of the disease by increasing the bacterial virulence and production of pyrogenic exotoxins [22]. Among a number of cell surface components expressed by group A streptococci, the major cell surface protein is M protein. The presence of this protein on a group A streptococcus correlates with its capacity to resist phagocytic killing. This is because M protein molecules bind to plasma fibrinogen and so inhibit complement activation and deposition of opsonic complement fragments on the bacterial cell [15]. Indeed, both a small wound and the intake of NSAIDs that might have triggered the clinical course of a potential streptococcal infection were present in the discussed patient; however, typical clinical symptoms of streptococcal toxic shock syndrome include sudden diffuse severe pain with fever, flu-like symptoms and a scarlet-like rash (in up to 10% of cases) [23]. Initial manifestation of abdominal pain and cholecystitis, followed by acute respiratory distress syndrome, renal and hepatic failure and disseminated intravascular coagulation is rare but has been reported [24]. Furthermore, group A streptococcus can induce acute, nonrheumatic myocarditis resembling acute myocardial infarction with ST-elevation [25]. Although no epidemiologic data are available that estimate the incidence of group A streptococcus-induced myocarditis, it may be responsible for more cases than previously thought.

Indeed, the history of a sore throat, fever and myocarditis strongly hints at group A streptococcal infection in the discussed patient. According to the literature, the mean latency of pharyngitis or tonsillitis and the onset of chest pain in patients with group A streptococcus-induced myocarditis ranges from 3 to 5 days [26–28]. Such latency would basically be compatible with the clinical course of our patient; however, he never complained of chest pain, which finally makes the diagnosis of group A streptococcus-induced myocarditis unlikely.

This finally leads to the discussion of leptospirosis, which is a zoonosis caused by spirochetes of the genus *Leptospira* [29]. Leptospirosis is endemic in tropical and temperate regions [30], but the disease has been recognized as an emerging global public health problem because of its endemic proportions and increasing incidence in both developing and developed countries [31, 32]. Leptospirosis presents with protean and nonspecific manifestations along with nonspecific findings in routine laboratory investigations, such as increases in CRP, CK, creatinine and transaminases, and thrombopenia [33]. Symptoms are usually a flu-like syndrome with fever, headache and myalgia that may resolve spontaneously; however, in some cases, the disease runs a severe course, leading to multiorgan failure with hemorrhage, hepatic, renal and pulmonary injury and septic shock [33, 34]. The mortality rate in leptospirosis ranges from 3% to 5% in severe cases and is primarily due to acute hepatic or renal failure, myocarditis, pulmonary hemorrhage and multiorgan failure [35, 36]. Leptospirosis is a biphasic disease characterized by an early phase (anicteric) lasting about one week and a delayed immune phase (icterohemorrhagic) in which most complications occur [29, 30]. This disease should be highly suspected whenever febrile patients who were previously healthy present with septic shock, acute renal and hepatic dysfunction and respiratory failure requiring intensive care [37] as presented in this case. Conjunctival suffusion was not reported in the discussed patient but would be an important clinical feature supporting the diagnosis of leptospirosis. Cardiac involvement with occurrence of acute myocarditis may be found in leptospirosis [38–40]; however, the frequency and extent of cardiac involvement in this disease are underreported and the pathophysiology is poorly understood. A variety of electrocardiographic changes may occur with atrial fibrillation, atrioventricular conduction blocks and nonspecific ventricular repolarization abnormalities being the most common. Electrolyte abnormalities are likely to contribute to electrocardiographic changes; direct effects on Na^+^, K^+^ and Cl^−^ transporters in the renal tubules have been postulated. Furthermore, histopathological changes from postmortem studies have shown myocardial inflammation and vasculitis in patients with cardiac involvement of leptospirosis [38]. Myocarditis usually occurs during the 5th and 7th day of leptospiral infections; significant left ventricular dysfunction is rare [35]. Fulminant leptospiral myocarditis requires early aggressive management including extracorporeal membrane oxygenation support if needed. Management of hemodynamic instability associated with leptospiral myocarditis is primarily supportive. Although yet unvalidated, the additional use of high-dose pulsed steroids may be beneficial [41].

There were many candidates on the differential diagnosis list in this patient, but in view of the entire constellation of findings, leptospirosis (Weil’s disease) seems to be the most likely final diagnosis. This should be confirmed by a microscopic agglutination test (MAT) revealing antibodies against leptospires.

### Dr. C. Wenisch’s diagnosis

Leptospirosis

## Discussion of case

### *Drs. K. Prein* and *U. Maierhofer:*

Autopsy showed edema and blood congestion in both lungs; clear yellowish effusions were present in the right (160 mL) and left (240 mL) pleural cavities. There was no evidence of pneumonia or pulmonary embolism. The bronchi were obstructed by yellowish-green mucus. The heart showed mild left ventricular hypertrophy (weight 330 g) and moderate dilatation, but no signs of myocardial infarction; the coronary arteries were unremarkable. Histology revealed mild infiltration with CD3-positive lymphocytes and CD68-positive macrophages, suggestive of mild myocarditis.

**Fig. 1:**
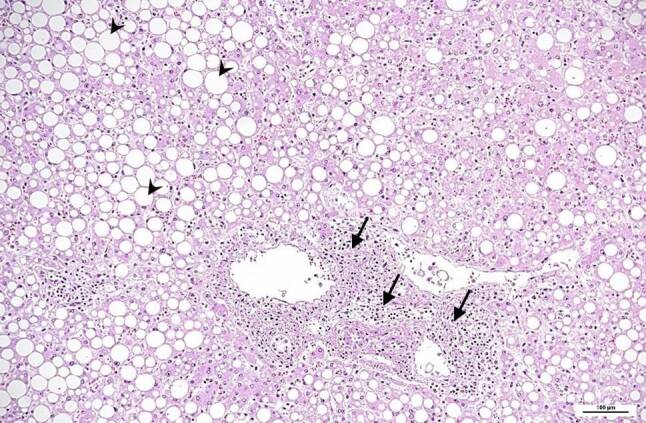
Liver histology: portal field with mild lymphocytic infiltration (*arrows*); massive, predominantly macrovesicular steatosis (*arrowheads*). Hematoxylin and eosin, original magnification 100 ×

**Fig. 2:**
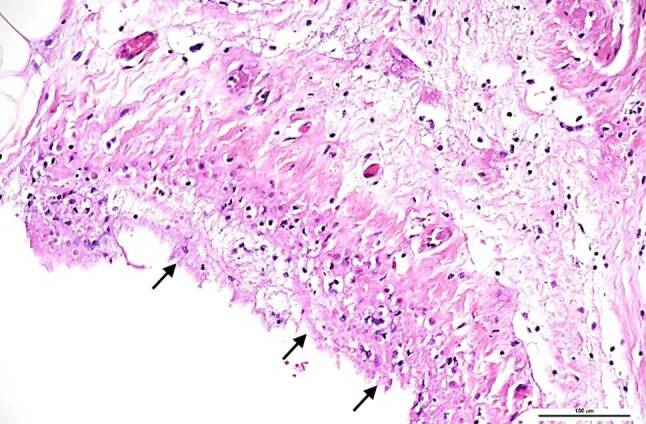
Serosa of the colon covered by fibrin interspersed with neutrophils (*arrows*). Hematoxylin and eosin, original magnification 200 ×

The peritoneal cavity contained 150 mL of turbid fluid. The liver was enlarged with a coarse border and multiple irregularly distributed bright yellow areas in the liver capsule (suspicious of ischemic areas). It weighed 2.5 kg. Histology showed massive steatosis (grade 3) with moderate inflammatory infiltration of the portal fields, and portal and focal septal fibrosis (stage 1), but no evidence of viral hepatitis (Fig. 1). The serosa of the bowel was covered and clotted with fibrin; histology showed massive infiltration with neutrophils and multiple bacteria, mainly of coccoid structure, which is typically found in fibrinous-putrid peritonitis (Fig. 2). The kidneys appeared normal on gross examination and displayed focal interstitial lymphocytic infiltration and mild arteriosclerosis on histology. The spleen, pancreas and stomach were macroscopically and histologically unremarkable.

Microbiological investigation revealed *Streptococcus anginosus* on the peritoneum. Stool cultures and tests for parasites including *Cryptosporidium parvum, Giardia lamblia* and *Entamoeba histolytica* were all negative.

### Dr. R. Krause:

After the patient had died a test result showing infection with *Leptospira copenhageni* (titer 1:400) became available. Infection is confirmed at a titer of > 1:100; results between 1:50 and 1:100 are borderline and should be repeated [42].

### Dr. K. Prein:

**Fig. 3:**
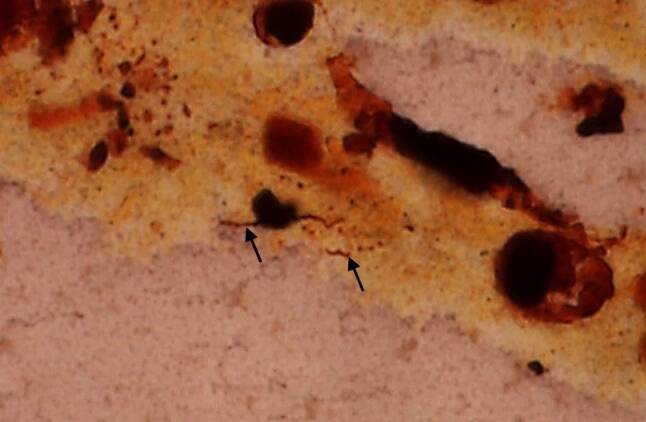
Spirochetal bacteria (*arrows*) consistent with leptospires within the peritoneal exudate. Warthin-Starry, original magnification 1000 ×

After the diagnosis of leptospirosis was confirmed we again investigated the peritoneal exudate using the Warthin-Starry stain and discovered spirochetal structures, i.e. leptospires (Fig. 3).

Even though the fulminant clinical course would let one expect more pronounced findings on autopsy we finally diagnosed multiorgan failure due to sepsis caused by leptospirosis.

### Dr. G.J. Krejs:

The patient presented with steatosis of the liver. Now the question is if this finding is somehow related to leptospirosis. Dr. Zollner is a hepatologist at the Medical University of Graz and will shortly comment on this.

### Dr. G. Zollner:

The most severe form of leptospirosis is called Weil’s disease; it is characterized by jaundice, renal dysfunction and hemorrhagic diathesis with a mortality rate of 40% and more [15, 31]. Although the jaundice can be profound it is usually not associated with severe hepatic dysfunction or necrosis [15]. Serum bilirubin is typically out of proportion to other values of liver function [43] and transaminases are only slightly raised [29]. Other nonspecific findings in leptospirosis include peripheral leukocytosis with a left shift, decreased platelets and impaired renal function [29].

In the discussed case, it is highly likely that hepatic steatosis had already been present before infection with *Leptospira copenhageni*; however, one should address the issue of whether pre-existing hepatic steatosis may have had a negative impact on the fulminant clinical course. Indeed, this may be true for patients with steatohepatitis in whom active inflammation and a decreased intestinal barrier can be found, but it seems very unlikely in patients with hepatic steatosis as the sole finding.

### Dr. C. Wenisch:

The clinical course of leptospirosis is variable and may be influenced by different factors. In this context, treatment with diclofenac should be discussed critically. Indeed, intake of this drug has been reported in the discussed case and also in a formerly published case of leptospirosis [33] with both patients developing fulminant disease with peritonitis, of which they died. Although valid data confirming an association between the intake of NSAIDs and mortality in leptospirosis are lacking, such data do exist for streptococcal myocarditis and streptococcal toxic shock syndrome [22]. Thus, one should be careful and probably not treat patients in whom leptospirosis is suspected with diclofenac.

### Dr. G.J. Krejs:

Diagnosis of leptospirosis may be challenging due to its varying clinical presentations. Among other infectious diseases, leptospirosis may also mimic acute abdomen. Henry Bockus wrote a now classic paper entitled “The internist looks at the acute abdomen” [44]. It contains several diagnoses, also referred to as pseudoacute abdomen or conditions in which laparotomy, which is the classical approach to acute abdomen, is a mistake. These are listed in Table 1.

**Table 1:** Causes of pseudoacute abdomen

Sickle cell crisis
Acute intermittent porphyria
Diabetic pseudoperitonitis
Spontaneous bacterial peritonitis
Lead poisoning
Addisonian crisis (adrenal crisis)
Acute glaucoma
Malaria, hantavirus infection and leptospirosis
Vasculitides
Familial Mediterranean fever
Proptosis (psychogenic bloating)

### Dr. R. Krause:

Leptospirosis is a global zoonotic disease caused by spirochetes of the genus *Leptospira* [45], which was first described in humans in 1886 by Adolf Weil [46]. In 1852, the disease had already been documented in dogs (Stuttgart disease) [47]. There are over 250 known pathogenic serovars, classified into serogroups, for which about 160 mammalian species have been identified as natural hosts. These include feral, semi-domestic and farm animals as well as pets as important reservoirs [48]. A predominant species of the genus *Leptospira* is *Leptospira interrogans*, which comprises some of the pathogenic serotypes causing leptospirosis; *Leptospira biflexa* comprise some of the saprophytic serotypes [49]. A more contemporary taxonomy classifies *Leptospira* into three phylogenetic lineages according to the degree of virulence of the species, namely saprophytic, intermediate and pathogenic [50]. Animal hosts remain more or less asymptomatic despite being infected. Bacteria are retained within the host’s renal tubules, where they thrive and multiply, and are occasionally shed via urine [51]. In a humid environment, microorganisms may survive for weeks [31]. Indirect infection by mucosal or percutaneous exposure to leptospires excreted into the environment (e.g. urine in water, soil or other contaminated material) is probably the main route of acquiring leptospirosis. A high incidence has been reported among people who are exposed to wet environments in their occupational activities [30, 32, 42]. Further, leptospirosis is more frequently found in people who are exposed to contaminated water during spare time activities such as swimming, canoeing, rafting, fishing and similar sports [30, 52, 53]. Gardening may be an underestimated category of risk exposure in Western countries [30]. Direct bacterial transmission from animals to humans is common among groups who handle animals and animal tissue, such as butchers, veterinarians, and cattle and pig farmers [31]. Recently, a 45-year-old veterinarian presented with cough and fever at our University Medical Center after pig urine had splashed in his face when he had accidently cut the bladder during meat inspection in a slaughterhouse. He was positive for leptospirosis and in the further disease course developed hemorrhagic pneumonia, which is frequently found in this disease due to tissue invasion of leptospires after hematogenous spread [54]. Leptospires are further able to evade the host immune system: They avoid complement-mediated killing through recruitment of host complement regulators, acquisition of host proteases that cleave complement proteins on the bacterial surface and by secretion of proteases that inactivate complement proteins in their surroundings [55]. Furthermore, leptospires may also directly evade macrophages and neutrophils until anti-leptospiral antibodies are produced [56]. It has been suggested that leptospires can hide within these immune cells and are released during apoptosis [57].

The symptoms of acute-phase leptospirosis include sudden onset of fever, myalgia and conjunctival suffusion; nausea, diarrhea, vomiting and chills may also be present [58]. The great majority of infections are either subclinical or mild and run a self-limiting anicteric course [30]. In about 10% of affected patients, infection leads to severe and possibly fatal Weil’s disease, which is characterized by hemorrhage, renal failure and jaundice caused by intrahepatic cholestasis and direct hepatocyte damage [29]. Hemorrhage can affect different organs; hyphema may occur in rare cases [59] due to autoimmune mechanisms [60]. More than 10^3^ leptospires per mL of blood prior to the introduction of antibiotics is associated with a higher risk of developing severe leptospirosis [61]. Leptospirosis has protean manifestations and mimics the clinical presentations of many other diseases. Since this disease can also cause severe acute respiratory distress syndrome (SARS), its diagnosis may be even more difficult at times of the pandemic caused by SARS coronavirus 2 (SARS-CoV-2) [62]. The broad clinical spectrum seen in leptospirosis is presumably the result of infection with different *Leptospira spp.* inducing different cellular and molecular mechanisms consequently leading to different disease expression; however, the details of these mechanisms remain to be elucidated.

Leptospirosis is more frequently found in tropical areas, but also occurs in temperate areas including Europe. In recent years, the disease has gained increasing attention as an emerging infectious disease of global importance [29, 63] with an increasing incidence in both developing and developed countries [31]. According to the estimates of the Leptospirosis Epidemiology Reference Group of the World Health Organization, approximately 1.03 million cases occur globally each year resulting in some 58,900 deaths [64]. In Austria, the annual epidemiological reports on communicable disease showed 8–11 cases of leptospirosis per year until 2010 [65]. With 24 cases, this number has more than doubled in 2019 [66]; however, data provide evidence of a high prevalence (29%) of antibodies against *Leptospira spp*. in Austrian male adults [67]. Most frequently serovar Canicola (16.5%), which is linked to dogs, and serovar Hardjo (12%), which is excreted by cattle, are found [51, 67]. Furthermore, data from southeast Austria and Upper Austria show that the vast majority of leptospirosis cases are autochthonously acquired and are associated with being exposed during activities in the woods and wet areas, as well as after contact with rodents [32, 68].

Different tests are available to diagnose leptospirosis. Serology is the most frequently used diagnostic tool. The microscopic agglutination test (MAT), which detects agglutinating antibodies in serum, is the reference standard test for serological diagnosis of leptospirosis with high sensitivity and specificity [69, 70]; however, MAT is limited by its subjectivity, and it requires the maintenance of live leptospires and a convalescent sample for conclusive results [71]. The standard criterion for a positive MAT is a titer of 1:400 or a fourfold increase in antibody titer in endemic countries, or a titer of 1:100 or above in nonendemic countries [72]. Besides MAT, rapid point-of-care IgM assays are available. Since IgM antibodies against leptospires become detectable during the first week of infection [29], these assays often become positive before MAT [69]. Molecular-based diagnostic testing is increasingly used for diagnosis of leptospirosis. Although quantitative PCR-based assays offer the ability to quantify the bacterial load in clinical specimens, the sensitivity (18–50%) is limited [71].

Leptospires can be isolated from blood or cerebrospinal fluid during the first 7–10 days of infection, and from urine during the 2nd and 3rd week of disease [73, 74]; however, culturing is difficult, insensitive and requires prolonged incubation (up to 3 months); the specific culture media are only available in few specialized laboratories [29]. Dark-field microscopy to see organisms in blood or urine is fraught with false positives and false negatives and is therefore not recommended [75].

Treatment of severe leptospirosis includes supportive care and use of appropriate antibiotics. Recommended regimens and doses are based on the severity of the disease. For mild disease, doxycycline, ampicillin or amoxicillin are preferred [76, 77], while for the treatment of severe leptospirosis, penicillin G, ampicillin or ceftriaxone are indicated [78]. Considering increasing antibiotic resistance and patients with penicillin allergy, the broad-spectrum third generation cephalosporins ceftriaxone and cefotaxime have been shown to be as effective as penicillin G in the treatment of severe leptospirosis [78, 79].

### Dr. C. Wenisch:

Leptospirosis presents with variable and nonspecific manifestations. Physicians should be aware of it and maintain a high index of suspicion when a previously healthy patient presents with fever, septic shock, acute renal and hepatic dysfunction, and respiratory failure requiring intensive care.

## Final diagnosis

Leptospirosis

## References

[1] Cooper LT. Clinical manifestations and diagnosis of myocarditis in adults. UpToDate. https://www.uptodate.com/contents/clinical-manifestations-and-diagnosis-of-myocarditis-in-adults?search=undefined&topicRef=130803&source=related_link. Accessed 10 Feb 2021

[2] Omar HR, Fathy A, Rashad R, Elghonemy M (2009). Acute perimyocarditis mimicking transmural myocardial infarction. Int Arch Med.

[3] Singer M, Deutschman CS, Seymour CW, Shankar-Hari M, Annane D, Bauer M, Bellomo R, Bernard GR, Chiche JD, Coopersmith CM, Hotchkiss RS, Levy MM, Marshall JC, Martin GS, Opal SM, Rubenfeld GD, van der Poll T, Vincent JL, Angus DC (2016). The Third International Consensus Definitions for Sepsis and Septic Shock (Sepsis-3). JAMA.

[4] Karzon DT, Edwards KM (1988). Diphtheria outbreaks in immunized populations. N Engl J Med.

[5] Clarke K, MacNeil A, Hadler S, Scott C, Tiwari TSP, Cherian T (2019). Global epidemiology of diphtheria, 2000–2017. Emerg Infect Dis.

[6] Amanna IJ, Slifka MK (2020). Successful vaccines. Curr Top Microbiol Immunol.

[7] Acosta AM, Moro PL, Hariri S, Tiwari TSP (2020). Diphtheria. Epidemiology and prevention of vaccine-preventable diseases. The pink book: course textbook.

[8] Jayashree M, Shruthi N, Singhi S (2006). Predictors of outcome in patients with diphtheria receiving intensive care. Indian Pediatr.

[9] Weinert LA, Araujo-Jnr EV, Ahmed MZ, Welch JJ (1807). The incidence of bacterial endosymbionts in terrestrial arthropods. Proc Biol Sci.

[10] Adem PV (2019). Emerging and re-emerging rickettsial infections. Semin Diagn Pathol.

[11] Gillespie JJ, Williams K, Shukla M, Snyder EE, Nordberg EK, Ceraul SM, Dharmanolla C, Rainey D, Soneja J, Shallom JM, Vishnubhat ND, Wattam R, Purkayastha A, Czar M, Crasta O, Setubal JC, Azad AF, Sobral BS (2008). Rickettsia phylogenomics: unwinding the intricacies of obligate intracellular life. Plos One.

[12] Carvalho R, Vazquez D, Silveira P, Lencastre L (2017). Rickettsiosis: a rare challenge in ICU. Intensive Care Med.

[13] Chmielewski T, Rudzka D, Fiecek B, Maczka I, Tylewska-Wierzbanowska S (2011). Case of TIBOLA/DEBONEL (tick—borne lymphadenopathy/Dermacentor spp.—borne necrosis—erythema—lymphadenopathy) in Poland. Przegl Epidemiol.

[14] Kushawaha A, Brown M, Martin I, Evenhuis W. Hitch-hiker taken for a ride: an unusual cause of myocarditis, septic shock and adult respiratory distress syndrome. BMJ Case Rep. 2013. 10.1136/bcr-2012-007155. 10.1136/bcr-2012-007155 PMC3603717 23314875

[15] Jameson JL, Fauci AS, Kasper DL, Hauser SL, Longo DL, Loscalzo J (2018). Harrison’s principles of internal medicine.

[16] Vanek NN, Kazi S, Cepero NM, Tang S, Rex JH (1996). Human ehrlichiosis causing left ventricular dilatation and dysfunction. Clin Infect Dis.

[17] Havens NS, Kinnear BR, Mató S (2012). Fatal ehrlichial myocarditis in a healthy adolescent: a case report and review of the literature. Clin Infect Dis.

[18] Dinges MM, Orwin PM, Schlievert PM (2000). Exotoxins of Staphylococcus aureus. Clin Microbiol Rev.

[19] McCormick JK, Yarwood JM, Schlievert PM (2001). Toxic shock syndrome and bacterial superantigens: an update. Annu Rev Microbiol.

[20] Wharton M, Chorba TL, Vogt RL, Morse DL, Buehler JW (1990). Case definitions for public health surveillance. MMWR Recomm. Rep..

[21] Kaul R, McGeer A, Low DE, Green K, Schwartz B (1997). Population-based surveillance for group A streptococcal necrotizing fasciitis: clinical features, prognostic indicators, and microbiologic analysis of seventy-seven cases. Ontario group A streptococcal study. Am J Med.

[22] Stevens DL, Tanner MH, Winship J, Swarts R, Ries KM, Schlievert PM, Kaplan E (1989). Severe group A streptococcal infections associated with a toxic shock-like syndrome and scarlet fever toxin A. N Engl J Med.

[23] Stevens DL (1995). Could nonsteroidal antiinflammatory drugs (NSAIDs) enhance the progression of bacterial infections to toxic shock syndrome?. Clin Infect Dis.

[24] Hung TY, Wang LY, Chen CT, Chen TJ (2005). Streptococcal toxic shock syndrome with initial manifestation of abdominal pain and cholecystitis. Acta Paediatr Taiwan.

[25] Aguirre JL, Jurado M, Porres-Aguilar M, Olivas-Chacon C, Porres-Muñoz M, Mukherjee D, Taveras J (2015). Acute nonrheumatic streptococcal myocarditis resembling ST-elevation acute myocardial infarction in a young patient. Proc.

[26] Talmon Y, Gilbey P, Fridman N, Wishniak A, Roguin N (2008). Acute myopericarditis complicating acute tonsillitis: beware the young male patient with tonsillitis complaining of chest pain. Ann Otol Rhinol Laryngol.

[27] Mokabberi R, Shirani J, Haftbaradaran MA, Go BD, Schiavone W (2010). Streptococcal pharyngitis-associated myocarditis mimicking acute STEMI. JACC Cardiovasc Imaging.

[28] Upadhyay GA, Gainor JF, Stamm LM, Weinberg AN, Dec GW, Ruskin JN (2012). Acute nonrheumatic streptococcal myocarditis: STEMI mimic in young adults. Am J Med.

[29] Bharti AR, Nally JE, Ricaldi JN, Matthias MA, Diaz MM, Lovett MA, Levett PN, Gilman RH, Willig MR, Gotuzzo E, Vinetz JM (2003). Peru-United States Leptospirosis Consortium. Leptospirosis: a zoonotic disease of global importance. Lancet Infect Dis.

[30] Levett P (2001). Leptospirosis. Clin Micorbiol Rev.

[31] Vijayachari P, Sugunan AP, Shriram AN (2008). Leptospirosis: an emerging global public health problem. J Biosci.

[32] Hoenigl M, Wallner C, Allerberger F, Schmoll F, Seeber K, Wagner J, Valentin T, Zollner-Schwetz I, Flick H, Krause R (2014). Autochthonous leptospirosis in South-East Austria, 2004–2012. Plos One.

[33] Scharfetter A, Mühlhans M, Payer S, Wenisch C (2004). Three cases of leptospirosis requiring intensive care. Eur J Clin Microbiol Infect Dis.

[34] Mikulski M, Boisier P, Lacassin F, Soupé-Gilbert ME, Mauron C, Bruyere-Ostells L, Bonte D, Barguil Y, Gourinat AC, Matsui M, Vernel-Pauillac F, Goarant C (2015). Severity markers in severe leptospirosis: a cohort study. Eur J Clin Microbiol Infect Dis.

[35] Pushpakumara J, Prasath T, Samarajiwa G, Priyadarshani S, Perera N, Indrakumar J (2015). Myocarditis causing severe heart failure—an unusual early manifestation of leptospirosis: a case report. BMC Res Notes.

[36] Trevejo RT, Rigau-Pérez JG, Ashford DA, McClure EM, Jarquín-González C, Amador JJ, de los RJO, Gonzalez A, Zaki SR, Shieh WJ, McLean RG, Nasci RS, Weyant RS, Bolin CA, Bragg SL, Perkins BA, Spiegel RA (1998). Epidemic leptospirosis associated with pulmonary hemorrhage-Nicaragua, 1995. J Infect Dis.

[37] Katz AR, Ansdell VE, Effler PV, Middleton CR, Sasaki DM (2001). Assessment of the clinical presentation and treatment of 353 cases of laboratory-confirmed leptospirosis in Hawaii, 1974–1998. Clin Infect Dis.

[38] Navinan MR, Rajapakse S (2012). Cardiac involvement in leptospirosis. Trans R Soc Trop Med Hyg.

[39] Boignard A, Bonadona A, Hamidfar R, Pavese P, Bouvaist H, Hammer L, Rey I, Schwebel C, Vanzetto G, Barnoud D (2006). Cardiogenic shock due to acute myocarditis complicating leptospirosis. Arch Mal Coeur Vaiss.

[40] Jayathilaka PGNS, Mendis ASV, Perera MHMTS, Damsiri HMT, Gunaratne AVC, Agampodi SB (2019). An outbreak of leptospirosis with predominant cardiac involvement: a case series. BMC Infect Dis.

[41] Khoo CY, Ng CT, Zheng S, Teo LY (2019). An unusual case of fulminant leptospiral myocarditis: a case report. Eur Heart J Case Rep.

[42] Hoffmeister B, Peyerl-Hoffmann G, Pischke S, Zollner-Schwetz I, Krause R, Müller MC, Graf A, Kluge S, Burchard GD, Kern WV, Suttorp N, Cramer JP (2010). Differences in clinical manifestations of imported versus autochthonous leptospirosis in Austria and Germany. Am J Trop Med Hyg.

[43] Edwards CN, Nicholson GD, Everard COR (1982). Thrombocytopenia in leptospirosis. Am J Trop Med Hyg.

[44] Bockus HL (1958). The internist looks at the acute abdomen. Gastroenterology.

[45] Resch G, Awad-Masalmeh M, Bakoss P, Jareková J (2007). Utility of phylogenetic studies in the identification of Leptospira strains. Epidemiol Infect.

[46] Weil A. Ueber eine eigenthümliche mit Milztumor, Icterus und Nephritis einhergehende acute Infectionskrankheit. Dtsch Arch Klin Med. 1886;39(14):209–232.

[47] Byrne R (1955). Canine leptospirosis and public health. Public Health Rep.

[48] European Centre for Disease Prevention and Control (ECDC). Factsheet about leptospirosis. https://www.ecdc.europa.eu/en/leptospirosis/factsheet. Accessed 10 Feb 2021.

[49] Thayaparan S, Robertson ID, Fairuz A, Suut L, Abdullah MT (2013). Leptospirosis, an emerging zoonotic disease in Malaysia. Malays J Pathol.

[50] Perolat P, Chappel RJ, Adler B, Baranton G, Bulach DM, Billinghurst ML, Letocart M, Merien F, Serrano MS (1998). Leptospira fainei sp. nov., isolated from pigs in Australia. Int J Syst Bacteriol.

[51] Goarant C (2016). Leptospirosis: risk factors and management challenges in developing countries. Res Rep Trop Med.

[52] Brockmann S, Piechotowski I, Bock-Hensley O, Winter C, Oehme R, Zimmermann S, Hartelt K, Luge E, Nöckler K, Schneider T, Stark K, Jansen A (2010). Outbreak of leptospirosis among triathlon participants in Germany, 2006. BMC Infect Dis.

[53] Radl C, Müller M, Revilla-Fernandez S, Karner-Zuser S, de Martin A, Schauer U, Karner F, Stanek G, Balcke P, Hallas A, Frank H, Fürnschlief A, Erhart F, Allerberger F (2011). Outbreak of leptospirosis among triathlon participants in Langau, Austria, 2010. Wien Klin Wochenschr.

[54] Wunder EA, Figueira CP, Santos GR, Lourdault K, Matthias MA, Vinetz JM, Ramos E, Haake DA, Picardeau M, Dos Reis MG, Ko AI (2016). Real-time PCR reveals rapid dissemination of Leptospira interrogans after intraperitoneal and conjunctival inoculation of hamsters. Infect Immun.

[55] Fraga TR, Isaac L, Barbosa AS (2016). Complement evasion by pathogenic Leptospira. Front Immunol.

[56] Banfi E, Cinco M, Bellini M, Soranzo MR (1982). The role of antibodies and serum complement in the interaction between macrophages and leptospires. J Gen Microbiol.

[57] Jin D, Ojcius DM, Sun D, Dong H, Luo Y, Mao Y, Yan J (2009). Leptospira interrogans induces apoptosis in macrophages via caspase-8- and caspase-3-dependent pathways. Infect Immun.

[58] Haake DA, Levett PN (2015). Leptospirosis in humans. Curr Top Microbiol Immunol.

[59] Langner-Wegscheider BJ, Krause R (2015). Images in clinical medicine. Hyphema. N Engl J Med.

[60] Faber NA, Crawford M, LeFebvre RB, Buyukmihci NC, Madigan JE, Willits NH (2000). Detection of Leptospira spp. in the aqueous humor of horses with naturally acquired recurrent uveitis. J Clin Microbiol.

[61] Tubiana S, Mikulski M, Becam J, Lacassin F, Lefèvre P, Gourinat AC, Goarant C, D’Ortenzio E (2013). Risk factors and predictors of severe leptospirosis in New Caledonia. PLoS Negl Trop Dis.

[62] Vogel N (2020). Leptospira – Zebra unter der „Coronaherde“ [Leptospira-Zebra under the “corona herd”. Internist.

[63] Soo ZMP, Khan NA, Siddiqui R (2020). Leptospirosis: Increasing importance in developing countries. Acta Trop.

[64] Costa F, Hagan JE, Calcagno J, Kane M, Torgerson P, Martinez-Silveira MS, Stein C, Abela-Ridder B, Ko AI (2015). Global Morbidity and mortality of leptospirosis: a systematic review. PLoS Negl Trop Dis.

[65] European Centre for Disease Prevention and Control (2011). Annual epidemiological report 2011. Reporting on 2009 surveillance data and 2010 epidemic intelligence data.

[66] Statistics Austria.. http://statistik.at/web_de/statistiken/menschen_und_gesellschaft/gesundheit/gesundheitszustand/anzeigepflichtige_krankheiten/022361.html. Accessed 12 Dec 2020.

[67] Poeppl W, Orola MJ, Herkner H, Müller M, Tobudic S, Faas A, Mooseder G, Allerberger F, Burgmann H (2013). High prevalence of antibodies against Leptospira spp. in male Austrian adults: a cross-sectional survey, April to June 2009. Euro Surveill.

[68] Windpessl M, Prammer W, Nömeyer R, Dinkhauser P, Wimmer L, Müller P, Gusenleitner J, Wallner M (2014). Leptospirosis and renal failure: a case series. Wien Klin Wochenschr.

[69] Cumberland P, Everard CO, Levett PN (1999). Assessment of the efficacy of an IgM-elisa and microscopic agglutination test (MAT) in the diagnosis of acute leptospirosis. Am J Trop Med Hyg.

[70] Cole JR, Sulzer CR, Pursell AR (1973). Improved microtechnique for the leptospiral microscopic agglutination test. Appl Microbiol.

[71] Agampodi SB, Matthias MA, Moreno AC, Vinetz JM (2012). Utility of quantitative polymerase chain reaction in leptospirosis diagnosis: association of level of leptospiremia and clinical manifestations in Sri Lanka. Clin Infect Dis.

[72] Faine S (1982). Guidelines for the the control of leptospirosis.

[73] Levett PN (2003). Usefulness of serologic analysis as a predictor of the infecting serovar in patients with severe leptospirosis. Clin Infect Dis.

[74] Weyant RS, Bragg SL, Kaufmann AF, Murray PR, Baron EJ, Pfaller MA, al (1999). Leptospira and leptonema. Manual of clinical microbiology.

[75] Vinetz JM (2001). Leptospirosis. Curr Opin Infect Dis.

[76] Takafuji ET, Kirkpatrick JW, Miller RN, Karwacki JJ, Kelley PW, Gray MR, McNeill KM, Timboe HL, Kane RE, Sanchez JL (1984). An efficacy trial of doxycycline chemoprophylaxis against leptospirosis. N Engl J Med.

[77] Guidugli F, Castro AA, Atallah AN (2000). Antibiotics for preventing leptospirosis. Cochrane Database Syst Rev.

[78] Suputtamongkol Y, Niwattayakul K, Suttinont C, Losuwanaluk K, Limpaiboon R, Chierakul W, Wuthiekanun V, Triengrim S, Chenchittikul M, White NJ (2004). An open, randomized, controlled trial of penicillin, doxycycline, and cefotaxime for patients with severe leptospirosis. Clin Infect Dis.

[79] Panaphut T, Domrongkitchaiporn S, Vibhagool A, Thinkamrop B, Susaengrat W (2003). Ceftriaxone compared with sodium penicillin g for treatment of severe leptospirosis. Clin Infect Dis.

